# MmpL Proteins in Physiology and Pathogenesis of *M. tuberculosis*

**DOI:** 10.3390/microorganisms7030070

**Published:** 2019-03-05

**Authors:** Geoff Melly, Georgiana E. Purdy

**Affiliations:** Department of Molecular Microbiology & Immunology, Oregon Health & Science University, Portland, OR 97239, USA; melly@ohsu.edu

**Keywords:** *Mycobacterium tuberculosis*, lipids, MmpL, lipid transport, cell envelope

## Abstract

*Mycobacterium tuberculosis* (*Mtb*) remains an important human pathogen. The *Mtb* cell envelope is a critical bacterial structure that contributes to virulence and pathogenicity. Mycobacterial membrane protein large (MmpL) proteins export bulky, hydrophobic substrates that are essential for the unique structure of the cell envelope and directly support the ability of *Mtb* to infect and persist in the host. This review summarizes recent investigations that have enabled insight into the molecular mechanisms underlying MmpL substrate export and the role that these substrates play during *Mtb* infection.

## 1. Introduction

**Figure 1:**
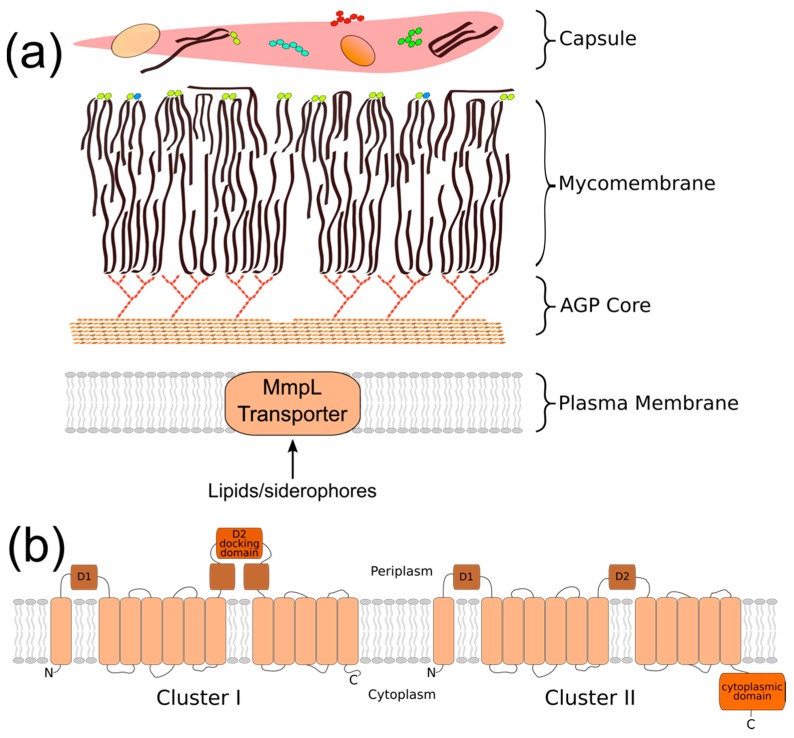
Mycobacterial membrane protein large (MmpL) proteins are topologically complex membrane proteins that export cell envelope components across the plasma membrane. (**a**) Cartoon representation of the mycobacterial cell envelope; (**b**) *M. tuberculosis* (*Mtb*) MmpL can be classified into two clusters based on the complexity of the D2 periplasmic domain and the presence of a cytoplasmic C-terminal domain. Cluster I = MmpL1/2/4/5/6/7/8/9/10/12; cluster II = MmpL3/11/13a/b.

The cell envelope of *Mycobacterium tuberculosis* (*Mtb*) is a key attribute of this bacterial pathogen that promotes survival and persistence in the face of host immune responses. This unique structure consists of four regions that define the border between the bacterial cytoplasm and the extracellular environment (Figure 1a). These are the: (1) plasma membrane, (2) arabinogalactan/peptidoglycan cell wall (AGP) core, (3) mycobacterial outer membrane (MOM, or mycomembrane), and 4) “outer layer” (OL, or capsule) [1]. Together, they form a robust physical barrier that contributes to the intrinsic resistance of *Mtb* against host antimicrobial defenses and limits the access of chemotherapeutics to the bacterial cytoplasm [2]. Additionally, certain cell envelope components directly interfere with aspects of host immunity to potentiate bacterial survival [3,4,5,6]. Thus, the cell envelope is a critical aspect of *Mtb* physiology and virulence. Investigating the biological processes that generate the cell envelope has the potential to greatly improve the treatment of TB disease.

The MmpL (mycobacterial membrane protein large) proteins are increasingly recognized for their importance in establishing the mycobacterial cell envelope. The primary role of the MmpL is to translocate complex, virulence-associated envelope lipids and siderophores across the plasma membrane to the periplasmic space. In the H37Rv strain of *Mtb*, there are 13 MmpL proteins (MmpL1-13) [7,8]. The majority of MmpL family members are predicted to be >100-kDa, and to consist of 11–12 transmembrane domains (TMD) and two periplasmic loop domains (D1/2) [8,9,10]. Exceptions include MmpL6, which is truncated to 42-kDa (five TMD), and MmpL13, which comprised two adjacent open reading frames, *mmpL13a* and *mmpL13b*, that are predicted to encode proteins of 32-kDa (four TMD) and 50-kDa (seven TMD), respectively [8,9].

Other species of mycobacteria harbor different numbers of MmpL proteins. *M. leprae*, often considered to possess a “minimal” mycobacterial genome as a result of reductive evolution, has five intact *mmpL* genes (*mmpL3*/*4*/*7*/*10*/*11*) as well as a similarly split *mmpL13a*/*b* locus [11,12]. These six MmpL proteins likely represent the core set of MmpL transporters, given that they exhibit a high degree of syntenic conservation in both slow growing (*Mtb*/*M. leprae*/*M. avium*/*M. marinum*) and rapidly growing mycobacteria (*M. smegmatis*/*M. abscessus*) [13]. On the other hand, *M. abscessus* possesses up to 31 putative MmpL transporters [11]. This illustrates the increased prevalence of genes coding for MmpL transporters in the genomes of rapidly growing, compared to slow growing, mycobacteria species [13]. *Mtb* is a pernicious mycobacterial human pathogen and H37Rv is the most widely used *Mtb* reference strain; therefore, we strive, where possible, to consider the MmpL and their contributions to virulence and pathogenicity in this context.

A subset of H37Rv *mmpL* genes (*mmpL1*,*2*,*4*,*5*) are accompanied by accessory *mmpS* (mycobacterial membrane protein small; *mmpS1*,*2*,*4*,*5*) open reading frames, which are each predicted to contain one to two TMD [7,10]. MmpS4 and -S5 proteins have been shown to interact with their cognate MmpL transporters to permit substrate extrusion [14]. Little is known about the other MmpS proteins and their potential roles in the transport of MmpL substrates. *rv2198c* was annotated as *mmpS3* due to partial homology with other *mmpS* genes, though it was not located in an operon with a cognate *mmpL* gene. Recently, it was shown to be a component of the mycobacterial division machinery and renamed *lamA* [15].

## 2. Phylogeny/Structure/Bioenergetics

The MmpL family members are traditionally categorized as belonging to the RND (resistance, nodulation, and cell division) superfamily of integral membrane permeases based on their topological organization [8,16]. Phylogenetic comparison has further classified the 10 “full-length” MmpL into two hydrophobe/amphiphile efflux (HAE) subfamilies of the RND superfamily. The MmpL1/2/4/5/8/10/12 belong to the HAE2 family of Gram-positive efflux pumps, while the MmpL3/7/11 are instead categorized into the HAE3 archaeal family [9,17]. Chim et al. proposed a two-cluster MmpL classification scheme based on predicted structural motifs (Figure 1b) [18]. MmpL cluster I comprises MmpL1/2/4/5/6/7/8/9/10/12, and is distinguished by the presence of a predicted docking domain in the periplasmic D2 loop region. MmpL cluster II comprises MmpL3/11 and the predicted MmpL13a/b fusion protein. It lacks the D2 periplasmic docking domain and possesses a substantial C-terminal cytoplasmic domain. Both classification schemes classify MmpL3 and MmpL11 separate from the other MmpL transporters. 

Many RND family proteins contribute to antibiotic resistance and stress-response in Gram-negative bacteria (reviewed in Reference [19]). The best characterized RND protein is AcrB (*E. coli*), which, together with a membrane fusion protein (MFP) and an outer membrane factor (OMF), functions as a multidrug efflux pump to extrude substrates through the periplasm to the extracellular environment [20,21,22,23,24]. The association of RND-MFP-OMF proteins to form an extrusion channel through the periplasm is paradigmatic in Gram-negative bacteria, with notable examples found in *P. aeruginosa* (MexAB/OprM) [25,26] and *C. metallidurans* (ZneABC) [27,28]. While *Mtb* exhibits pseudo-Gram-negative membrane organization, it remains to be determined if the MmpL interact with periplasmic/outer membrane proteins to form a similar tripartite export apparatus that would enable efficient translocation and proper localization of their substrates. Periplasmic lipoproteins such as LprG and LppX are required for the proper surface expression of some cell envelope components, suggesting that these proteins may be functioning as MFPs [29,30]. However, these lipoproteins have not been shown to interact with any MmpL transporters. MmpS proteins may be candidate MFPs given that MmpS4/5 interact with MmpL4/5 and are required for the transport of their siderophore substrates. However, only some MmpL transporters have cognate MmpS proteins, and MmpL-MmpS protein–protein interactions have not been demonstrated for MmpS1/2. CpnT has been described as an *Mtb* outer membrane porin, and may be a putative OMF [31,32]. How MmpL substrates traverse the periplasmic space and cell wall core en route to the mycomembrane remains an area of active inquiry. 

The crystal structures of the *apo*- and inhibitor-bound *M. smegmatis* MmpL3 orthologue were recently reported, representing an important advancement in the field [33]. Notably, MmpL3 was observed to be monomeric, a finding that was corroborated by native protein electrophoresis. This differs from most known bacterial RND permeases, which typically associate as either homodimers or homotrimers [26,34,35]. Indeed, a previous structural study of the purified Corynebacterial MmpL3 orthologue CmpL1, via size exclusion chromatography and negative staining electron microscopy (EM), indicated homotrimeric association [36]. A subsequent MmpL3 homology structure, modeled on the EM-refined CmpL1 homology model, suggested that *Mtb* MmpL3 also associates as a homotrimer. Additional structures and analyses are needed to reconcile these conflicting observations and determine whether the functional MmpL transporter units differ by species and if all MmpL proteins are monomeric.

The transmembrane region of *apo*-MmpL3 exhibited pseudo-two-fold symmetry, with the central TM4 and TM10 domains hydrogen-bonded to each other via a pair of Asp-Tyr dyads [33]. The periplasmic region was observed to contain three openings leading to a central cavity, which are presumably important for substrate extrusion. Individually, the structure of each periplasmic MmpL3 D1 and D2 subdomain broadly resembled that of the isolated *Mtb* MmpL11 D2 domain, obtained by Chim et al. [18]. This work demonstrated a homology between the MmpL11 D2 domain and the periplasmic transporter/porter subdomains of the RND permeases MexB and ZneA. Furthermore, the MmpL11 D1 and D2 periplasmic domains were shown to interact with each other. This was also the case for the MmpL3 D1 and D2 periplasmic domains, suggesting that intra- or inter-MmpL interactions occur in the periplasm [18].

The proton motive force (PMF) is understood to be the underlying energetic basis for most RND-mediated substrate transport [24,37,38]. RND proteins generally function as antiporters, coupling proton translocation across the inner membrane to conformational changes that result in substrate extrusion [39,40]. A suite of conserved residues found in TM4 and TM10 are critical for proton transfer down the membrane electrochemical gradient [38,41,42]. Multiple sequence alignments of the *Mtb* H37Rv MmpL identified similarly conserved regions in MmpL TM4/10 [43]. These include the central Asp/Tyr-containing TM4/10 segments observed in the *M. smegmatis* MmpL3 crystal structure, strongly suggesting that the core of each MmpL monomer is responsible for proton translocation [33]. Indeed, mutation of either the TM10 D640 or Y641 residues completely abrogated MmpL3 function [43], while mutation of the homologous Y610 residue likewise resulted in cessation of MmpL11-mediated lipid transport (Purdy Lab, unpublished data). Furthermore, several ostensible chemical inhibitors of MmpL3 appear to function by non-specifically disrupting the PMF [44]. Others, including the developmental therapeutic drug SQ109, directly bind MmpL3 in the putative proton translocation channel defined by TM4/10 [33]. However, the observation that SQ109 failed to inhibit MmpL3-mediated substrate transport in a spheroplast-based assay raises questions about whether this interaction is sufficient to abrogate MmpL3 transporter activity [45]. Regardless of the exact mechanism of action of SQ109, the data strongly suggest that MmpL substrate transport is energetically dependent on the PMF, like that of most other RND permeases. 

## 3. Roles in Virulence

**Figure 2:**
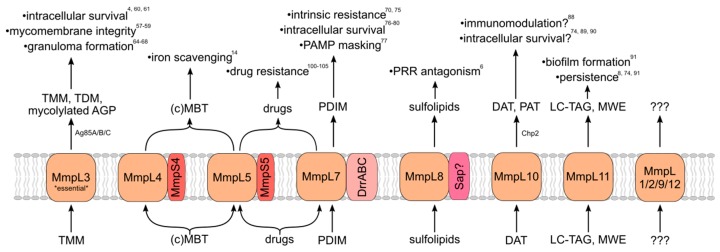
MmpL transporters export substrates that are important for virulence and pathogenicity. MmpL proteins and membrane-associated transport partners, exported substrates, and contributions to virulence/pathogenicity of the mature exported substrates. TMM/TDM—trehalose mono/di-mycolate; AGP—arabinogalactan/peptidoglycan complex; (c)MBT—(carboxy)mycobactin; PDIM—phthiocerol dimycocerosate; PAMP—pathogen-associated molecular pattern; PRR—pattern recognition receptor; DAT/PAT—di/poly-acyl trehalose; LC-TAG—long-chain triacylglycerol; and MWE—mycolate wax ester.

MmpL proteins indirectly contribute to virulence via the transport of substrates that directly influence bacterial survival in the human host when properly localized. Most, but not all, identified MmpL substrates are virulence-associated envelope lipids (Figure 2). MmpL-transported lipids are incorporated into both leaflets of the mycomembrane, from which *Mtb* derives much of its intrinsic resistance and immunomodulatory capacity. Other MmpL substrates are important for responding to environmental and nutrient stress. How MmpL substrates influence *Mtb* virulence is therefore essential for understanding the importance of the MmpL protein family to the pathogenic lifestyle of *Mtb*.

### 3.1. MmpL3 (TMM)

MmpL3 (Rv0206c) is responsible for transporting trehalose monomycolate (TMM), and is the only MmpL for whom transporter activity has been directly determined via biochemical assay [45]. TMM transport by MmpL3 has also been demonstrated indirectly, based on the observation that genetic or chemical ablation of MmpL3 activity reduces cell envelope mycolylation [46,47]. Mycolic acids are unique, long chain (C60-90) α-alkyl β-hydroxy fatty acids that are synthesized in the bacterial cytoplasm and esterified to the disaccharide trehalose to generate TMM (reviewed in References [48,49]). Once exported, mycolic acid transfer in the cell envelope is catalyzed by the members of the secreted antigen 85 mycolyltransferase complex (Ag85A/B/C, Rv3804c/1886c/0129c) [50,51,52]. When esterified to the non-reducing ends of the cell wall AGP core, mycolic acids constitute the inner leaflet of the mycomembrane [53,54,55]. Additionally, mycolic acid transfer between molecules of TMM generates trehalose dimycolate (TDM), an abundant and important virulence-associated free lipid [50,51,52]. 

Genetic approaches to knock out *mmpL3* have been uniformly unsuccessful; however, conditional knockdown and chemical inhibition strategies revealed that reduction or inhibition of MmpL3 caused accumulation of TMM in the cytoplasm and a reduction of cell envelope mycolylation [8,46,56,57,58]. MmpL3 is therefore the only essential MmpL protein, underscoring the importance of mycolic acid export to *Mtb* physiology. These observations also highlight MmpL3 as an attractive drug target [57,59]. 

Apart from their importance to the covalently-anchored inner leaflet of the mycomembrane, mycolic acids are also an essential component of TDM, perhaps the quintessential *Mtb* virulence-associated glycolipid [60,61]. TDM was originally described as a “cord factor,” referring to the tendency of mycobacteria harboring TDM to aggregate about their long axes to form “cords” of bacteria [62,63]. It plays an extremely important role in the infectious context by promoting *Mtb* survival in the macrophage through inhibiting phagosomal maturation via recruitment of the host pattern recognition receptor Mincle during Fcγ-R mediated phagocytosis [4]. In the host, purified TDM induces granuloma formation, also in a Mincle-dependent manner [64,65,66,67,68].

### 3.2. MmpL7 (PDIM)

MmpL7 (Rv2942) is associated with transport of phthiocerol dimycocerosates (PDIM), a family of long-chain β-diols esterified with polymethyl-branched fatty acids [69]. Interestingly, PDIM translocation is also dependent on the presence of the DrrABC (Rv2936-8) transporter in addition to MmpL7, a unique requirement for the transport of MmpL substrates [70]. Once exported into the periplasm, the proper surface localization of PDIM is mediated by the lipoprotein LppX (Rv2945c), potentially via a direct protein–lipid interaction [29]. 

The genes responsible for PDIM biosynthesis and transport are clustered together in the *Mtb* genome [71]. PDIM production occurs in the cytoplasm, and is likely spatially and temporally coordinated with MmpL7-mediated transport [72,73]. This model was suggested by an observed protein–protein interaction between the D2 domain of MmpL7 and the PDIM biosynthetic polyketide synthase PpsE (Rv2935) in a two-hybrid system [73]. This result was surprising since MmpL topology predictions strongly suggest that the D2 domain projects into the periplasm. Because PpsE is localized in the cytoplasm, this interaction could suggest that MmpL topology is fundamentally dissimilar to that of other RND family proteins, or, as suggested by the authors, that the D2 domain is able to access the cytoplasm, possibly through a pore formed by the MmpL7 TM domains. Whether this activity might be a shared feature of the *Mtb* MmpL or unique to MmpL7 is not known. 

MmpL7 is required for virulence in mouse models of infection due to its role in PDIM transport [8,74]. PDIM plays multiple roles in the virulence of *Mtb*. The highly hydrophobic nature of PDIM limits the permeability of the *Mtb* cell envelope and contributes to intrinsic resistance against antimicrobial compounds [70,75]. PDIM also promotes the uptake of *Mtb* by permissive macrophages [76,77], and appears to enable phagosomal escape upon internalization [76,77,78,79,80]. Additionally, PDIM contributes to *Mtb* virulence by masking the recognition of pathogen associated molecular patterns by host innate immune receptors [77].

### 3.3. MmpL8 (Sulfolipids)

MmpL8 (Rv3823c) transports cell envelope sulfolipids [81,82]. Sulfolipids consist of a sulfated trehalose tetra-acylated with straight chain and multiple polymethyl-branched fatty acids [83,84]. The diacylated SL_1278_ precursor is synthesized in the cytoplasm through the sequential esterification of sulfated trehalose with palmitate and (hydroxy)phthioceranoate by the polyketide synthase associated proteins PapA2 (Rv3820c) and PapA1 (Rv3824c), respectively [84]. Two additional (hydroxy)phthioceranoate additions are performed by the cytoplasmic membrane-associated acyltransferase Chp1 (Rv3822) to generate mature tetra-acylated sulfolipid-1 (SL-1), which is subsequently translocated by MmpL8 [85]. The integral membrane protein Sap (Rv3821), while not absolutely required for SL-1 transport, appears to enhance surface expression of SL-1. It is suggested that Sap interacts with MmpL8 and the sulfolipid biosynthetic enzymes to coordinate biosynthesis and transport [85]. 

Sulfolipids contribute to *Mtb* virulence by inhibiting activation of the host pattern recognition receptor toll-like receptor 2, thereby limiting the innate immune response to *Mtb* [6]. This immunomodulation is contingent upon proper localization of sulfolipids in the mycobacterial cell envelope. MmpL8-deficient *Mtb* exhibits attenuated growth and pathogenesis in mouse infection models [8,74,81,82].

### 3.4. MmpL10 (Di/Poly-Acyltrehaloses)

MmpL10 (Rv1183) translocates diacyltrehaloses (DAT) across the plasma membrane, where they are further acylated to generate penta-acyltrehaloses (PAT) [86]. DAT biosynthesis is initiated in the cytoplasm by the esterification of a straight chain fatty acid to the 2-position of trehalose by the acyltransferase PapA3 (Rv1182), which is likely also responsible for incorporation of a polymethyl-branched fatty acid at the 3-position [87]. MmpL10-mediated transport of DAT to the periplasm allows the transfer of additional polymethyl-branched fatty acid moieties between DATs by the periplasmic acyltransferase Chp2 (Rv1184c) to generate PAT [86]. The topological discontinuity of PAT biosynthesis is reminiscent of TDM biosynthesis, with transport of a cytoplasmic precursor to the periplasm where additional biosynthetic enzymes operate. 

The contribution of DAT/PAT to *Mtb* virulence is not completely understood. Purified DAT inhibited T-cell proliferation and production of proinflammatory cytokines by macrophages in vitro [88]. MmpL10 did not appear to contribute to *Mtb* virulence in a mouse aerosol infection model, implying that its substrates are dispensable for a typical *Mtb* infection [8]. However, another study found that an *mmpL10* mutant was attenuated in an intravenous mouse infection [74]. Perplexingly, a different study showed that a DAT/PAT-deficient strain of *Mtb* more readily infected macrophages and was hypervirulent in an intravenous, but not a respiratory, mouse infection model [89]. Finally, a recent study found that an *Mtb* DAT/PAT mutant was attenuated only in the absence of PDIM, leading the researchers to conclude that these methyl-branched cell envelope lipids may be functionally redundant [90]. 

### 3.5. MmpL11 (Long-Chain TAG/Mycolate Wax Esters)

MmpL11 (Rv0202c) transports long-chain triacylglycerols (LC-TAG) and mycolate wax esters (MWE) in *Mtb* [91]. These lipids are of particular importance during biofilm formation. Since *Mtb* in biofilms is phenotypically drug tolerant, LC-TAG and MWE may contribute to the extensive drug treatment regimens necessary to cure TB disease [92]. Furthermore, these lipids may play a role in persistence in the host. *Mtb mmpL11* mutants are attenuated for survival in an in vitro granuloma model and during long-term infections in mice, suggesting that MmpL11 lipid substrates are required for the maintenance of long-term *Mtb* infection [8,74,91]. 

### 3.6. MmpL4/5 (Mycobactin/Carboxymycobactin)

MmpL4 and MmpL5 are unique in that they do not transport a cell envelope lipid, but instead export mycobacterial siderophores. Iron is critical for many cellular processes and iron restriction is a common strategy used by hosts to combat infection. *Mtb* produces two siderophores to scavenge iron from the environment: lipophilic mycobactin (MBT) and hydrophilic carboxymycobactin (cMBT). The MmpL4/5 transporters (Rv0450c/0676c) and their cognate MmpS4/5 accessory proteins (Rv0451c/0677c) coordinate biosynthesis and transport of these siderophore substrates [14]. MmpL4 and MmpL5 are at least partially redundant, given that single mutants of either *mmpS4* or *mmpS5* are not affected in iron-limited culture conditions. However, an *mmpS4*/*5* double mutant exhibited a significant growth defect in low-iron culture, failed to produce and export siderophores, and was markedly less virulent in a mouse infection model compared to wild-type *Mtb* [14]. Interestingly, a genetic method showed that MmpL5 was capable of interacting with either MmpS4 or MmpS5, whereas MmpL4 was only capable of interacting with MmpS4 [14]. 

Siderophore export likely depends on MmpL/MmpS interactions in the periplasm. For MmpL4, this was suggested by the fact that the MmpL4 D1 periplasmic region co-precipitates with the periplasmic region of MmpS4 [14]. Another group demonstrated, via molecular dynamics simulations, that MBT is taken up from the cytoplasm by MmpL5, and that the periplasmic interaction between MmpL5 and MmpS5 was likely required for MBT release into the periplasm [93]. While in vitro models of iron sensitivity have suggested that the MmpL/S4 and S5 systems are functionally redundant, an *mmpL4* single mutant was attenuated in a mouse model of infection [8]. However, another group observed growth attenuation of *mmpL5* mutants in the lungs of infected mice after intravenous inoculation [74]. Interestingly, the same study reported a reduction in *mmpL4* mutant growth in the spleen compared to wild-type *Mtb*. Combined, these observations imply that the two different siderophore export systems have differing levels of importance depending on the infection context. 

### 3.7. Other MmpL Transporters

The substrates and functions of the *Mtb* MmpL1/2/6/9/12/13a/b transporters have not yet been conclusively identified. However, these transporters are not as critical as the aforementioned MmpL for virulence of *Mtb* as assessed by the mouse model of infection [8]. Nevertheless, some clues as to the functions of these transporters can be derived from the genomic contexts of their loci, and through comparison with orthologous proteins in other mycobacterial species. 

The genes encoding MmpL1 (Rv0402c) and MmpL12 (Rv1522c) are both located near *pks* and *fad* genes, implying that they transport complex glyco- or polyketide lipids similar to TMM or PDIM [11]. Indeed, the exported substrate of MAB_0855, a putative *M. abscessus* orthologue of MmpL12, was recently identified as a glycosyl diacylated nonadecyl diol, suggesting that the substrate of MmpL12 in *Mtb* is a heretofore undescribed complex cell envelope glycolipid [94]. 

Very little is known about MmpL2 (Rv0507). The genomic context of the *mmpL2* gene does not give any clues as to the nature of its putative substrate. MmpL2 is dispensable during pulmonary models of infection, and there are conflicting reports regarding its relevance during intravenous infection [8,71,74]. MmpL9 (Rv2339) is similarly mysterious. It appears to be involved in the inhibition of phagosomal maturation; however, loss of this capability did not impact the survival of a ∆*mmpL9* mutant strain of *Mtb* in ex vivo infections [95]. Another study observed increased susceptibility of an *mmpL9* mutant to in vitro oxidative stress [96]. Despite these observations, *mmpL9*-deficient *Mtb* is still able to replicate effectively in mouse infection models [8,74].

MmpL13a/b (Rv1145/1146) is a unique case. An intact full-length MmpL13 orthologue was found in both *M. bovis* and *M. canettii*, suggesting that the gene split occurred during the evolution of modern *Mtb* [9]. Interestingly, these genes appear to be separate, functional open reading frames in H37Rv *Mtb*, whereas they are pseudogenes in *M. leprae*, implying that the *mmpL13* gene is currently undergoing a pseudogenization process in modern *Mtb*. 

As mentioned, MmpL6 (Rv1557) is truncated relative to the other MmpL in H37Rv *Mtb*. This likely occurred during the evolution of modern circulating *Mtb* strains, during which its cognate *mmpS6* gene was completely lost [97]. A recent paper demonstrated that an un-truncated “ancestral” version of the *mmpL*/*S6* operon conferred oxidative stress resistance to “modern” *Mtb* lineages [98]. However, it is not known whether this increased resistance is linked to substrate export or to some other mechanism. Production of reactive oxygen species is a well-described defense mechanism of innate immunity (reviewed in Reference [99]). Thus, it is somewhat perplexing how this ostensibly beneficial means of intrinsic resistance was lost during *Mtb*-host co-evolution. 

### 3.8. MmpL as Drug Exporters

In addition to transporting their endogenous cell envelope substrates, certain MmpL transporters may also act as drug efflux pumps. This has been most convincingly shown for the MmpL5/S5 transporter system in both *Mtb* and nontuberculous mycobacteria (NTM). Increased resistance to azole drugs, as well as to clofazimine and bedaquiline, can arise through mutations in *rv0678*, a transcriptional repressor that inhibits expression of *mmpL5*/*S5* [100,101]. Altering the repressive ability of Rv0678 results in over-expression of *mmpL5*/*S5* and resistance to the aforementioned drugs via drug efflux. Similarly, mutations in the transcriptional repressor of an MmpL5/S5 orthologue also augmented resistance to clofazimine and bedaquiline in the clinically important NTM species *M. abscessus* [102]. De-repression of a different MmpL5/S5 orthologue in *M. abscessus* likewise conferred increased resistance to thioacetazone-based chemotherapeutics [103]. In addition to MmpL5/S5, MmpL7 has also been implicated in drug efflux. The upregulated expression of *mmpL7* in response to isoniazid treatment increases mycobacterial resistance to this important frontline TB antibiotic through direct export [104,105].

## 4. MmpL Regulation

### 4.1. Transcriptional Regulation

A number of transcriptional regulators that bind to *mmpL* genomic regions were identified via ChIP-seq [106]. These include Rv0302, Rv0678, Rv1816, and Rv3249c. Further studies revealed that these transcriptional regulators modulate their activity by binding fatty acid ligands, implying that *Mtb* alters its cell wall in response to metabolic cues [107,108,109,110,111]. This is particularly relevant during infection because *Mtb* metabolizes fatty acids in the host [107,112]. Rv0302 is a TetR-family transcriptional regulator that binds in the intragenic regions of *mmpL1*/*2*/*7*/*9*, upstream of *mmpL3* and in the promoters of *mmpL6* and *mmpL11* [106,109]. Upon binding palmitic acid, the Rv0302 dimer dissociates from its target DNA [109]. Rv3249 is also a TetR-like transcription factor that binds the promoters of *mmpS1* and *mmpL3*, and inside *mmpL11*. It also releases from its target DNA sequences upon binding palmitic acid [110]. Rv1816 binds to promoter and intergenic regions of *mmpL3*, *mmpL7*, and *mmpL11* [110]. Additionally, it binds *kasA*, which is involved in mycolic acid biosynthesis [113]. Thus, Rv1816 represents a single point of control at which production and export of cell envelope components may be transcriptionally regulated. Rv1816 binds two fatty acid ligands, lauric acid and palmitic acid; however, palmitic acid was the only ligand that reduced DNA binding activity [110]. Rv0678 is a member of the MarR family of transcriptional regulators that binds the promoter regions of *mmpL2*, *mmpL4*/*S4*, and *mmpS5* as a dimer [111]. The binding of 2-stearoylglycerol by Rv0678 reduced its ability to interact with target sequences [111]. 

Combined, these observations suggest that *Mtb* monitors its metabolic state and adjusts its cell wall composition in response to metabolic cues. This would presumably enable *Mtb* to better persist in the face of the host immune response during the course of establishing an infection. 

### 4.2. Post-Translational Regulation

MmpL transporter function may also be regulated through post-translational modification. A likely means of MmpL regulation is via phosphorylation by serine/threonine protein kinases (STPKs). *Mtb* has 11 STPKs, nine of which contain a cytoplasmic kinase domain, a transmembrane domain, and an extracellular sensor domain (reviewed in Reference [114]). STPK-mediated phospho-regulation of protein function is therefore an immediate way to alter cellular processes in response to external stimuli. 

MmpL7 is a potential substrate of the PknD kinase [115]. Surprisingly, the identified phospho-residue lies in the predicted D2 periplasmic loop region, which would not be expected to interact with the cytoplasmic kinase domain of PknD. However, as mentioned above, the MmpL7 D2 domain may access the cytoplasm [73]. How phosphorylation affects the MmpL7 transporter function has not been demonstrated.

MmpL3 and MmpL11 have substantial C-terminal cytoplasmic domains that are likely sites of phospho-regulation by mycobacterial STPKs. Indeed, both MmpL3 and MmpL11 were found to be phosphorylated in a comprehensive survey of *Mtb* phospho-proteins [116]. MmpL3 had extensive C-terminal phosphorylation, with multiple phospho-residues identified in a variety of culture and in vitro stress conditions. However, the MmpL11 C-terminus was only phosphorylated at a single residue when grown with acetate as the sole carbon source, suggesting that phosphorylation of MmpL proteins occurs in response to metabolic cues [116]. Future studies should investigate the effects of phosphorylation on MmpL transporter activity.

## 5. Conclusions

The MmpL transporters are an important family of inner membrane transporters in *Mtb*. Their substrates make immense contributions to *Mtb* virulence and pathogenicity. MmpL3-transported mycolic acids are an essential part of the mycomembrane and confer much of *Mtb*’s intrinsic resistance to the antimicrobial defenses of innate immunity. Other MmpL substrates such as PDIM, sulfolipids, and the acylated trehaloses modulate host immunity in subtler ways. Storage lipids exported by MmpL11 enable long-term persistence in the face of the adaptive immune response. Siderophore transport by MmpL4 and MmpL5 and their cognate MmpS proteins permits *Mtb* to acquire essential iron in the restrictive environment of the host. Therefore, this relatively small family of transporters plays a substantial role in supporting *Mtb*’s specialization as a devastating human pathogen.
